# Capturing the wide variety of impaired fracture healing phenotypes in Neurofibromatosis Type 1 with eight key factors: a computational study

**DOI:** 10.1038/srep20010

**Published:** 2016-01-29

**Authors:** A. Carlier, H. Brems, J. M. A. Ashbourn, I. Nica, E. Legius, L. Geris

**Affiliations:** 1 Biomechanics Section, KU Leuven, Celestijnenlaan 300 C, PB 2419, 3000 Leuven, Belgium; 2 Prometheus, Division of Skeletal Tissue Engineering, KU Leuven, O&N 1, Herestraat 49, PB 813, 3000 Leuven, Belgium; 3 Biomechanics Research Unit, University of Liège, Chemin des Chevreuils 1 – BAT 52/3, 4000 Liege 1, Belgium; 4 Department of Human Genetics, KU Leuven, O&N 1, Herestraat 49, PB 602, 3000 Leuven, Belgium; 5 Department of Engineering Science, University of Oxford, Parks Road, Oxford OX1 3PJ, U.K; 6 St Edmund Hall, University of Oxford, Queen’s Lane, Oxford OX1 4AR, U.K

## Abstract

Congenital pseudarthrosis of the tibia (CPT) is a rare disease which normally presents itself during early childhood by anterolateral bowing of the tibia and spontaneous tibial fractures. Although the exact etiology of CPT is highly debated, 40–80% of CPT patients are carriers of a mutation in the *Neurofibromatosis Type 1* (*NF1*) gene, which can potentially result in an altered phenotype of the skeletal cells and impaired bone healing. In this study we use a computational model of bone regeneration to examine the effect of the *Nf1* mutation on bone fracture healing by altering the parameter values of eight key factors which describe the aberrant cellular behaviour of *Nf1* haploinsufficient and *Nf1* bi-allelically inactivated cells. We show that the computational model is able to predict the formation of a hamartoma as well as a wide variety of CPT phenotypes through different combinations of altered parameter values. A sensitivity analysis by “Design of Experiments” identified the impaired endochondral ossification process and increased infiltration of fibroblastic cells as key contributors to the degree of severity of CPT. Hence, the computational model results have added credibility to the experimental hypothesis of a genetic cause (i.e. *Nf1* mutation) for CPT.

Congenital pseudarthrosis of the tibia (CPT) is a rare disease which normally presents during early infancy. It is characterised by anterolateral bowing of the tibia and develops into spontaneous fractures in the distal third of the tibia^1^. Usually the bone regeneration is insufficient and a pseudarthrosis is formed at the fracture site. Conventionally, CPT is treated surgically by the physical excision of the abnormal bone tissue (also described as the ‘hamartoma’) and internal or external fixation^1^. Failure to maintain a bony union is however common, resulting in multiple revision surgeries and occasionally amputation of the lower leg.

The exact etiology of CPT is still highly debated. However, both the fibrous hamartoma as well as the pathologically thick periosteum are believed to prevent the formation of a bony union due to mechanical interposition and hampered vascularisation^2^. Also, 40-80% of CPT patients are carriers of a mutation in the *NF1* gene whereas the incidence of CPT is less than 4% in patients with a *NF1* gene mutation^3^,^4^,^5^ (note that in a clinical setting rigorous genetic screening is often lacking which might lead to undetected *NF1* mutations in CPT patients). It has been postulated that a double inactivation of the *NF1* gene is necessary for the development of a pseudarthrosis^6^,^7^, and that the relative proportion of *NF1* haploinsufficient cells and *NF1* bi-allelically inactivated cells in the pseudarthrosis region will determine the healing outcome. Hence, the altered phenotype of mutated skeletal cells may also contribute to the observed impaired bone healing in patients with a *NF1* gene mutation (Fig. 1A). The *NF1* gene encodes neurofibromin, an ubiquitous protein expressed in osteoblasts, osteoclasts, chondrocytes, fibroblasts and endothelial cells^1^,^8^. Neurofibromin negatively regulates the activity of Ras, a protein involved in cellular proliferation and differentiation^1^,^2^. One of the cellular deficiencies is the recurrent invasion and increased proliferation of fibrous lesion cells, thereby physically preventing bony bridging^1^. In addition, the matrix production of the lesionous chondrocytes is affected which hampers endochondral bone repair^1^. Furthermore, the mutated osteoprogenitor cells show inferior osteogenic differentiation while excessive generation of osteoclasts may compromise any attempt at bone repair^1^,^9^. Finally, lesionous endothelial cells may interfere with normal bone healing through an altered blood supply^1^,^10^.

Clearly, an improved understanding of the role of *NF1* haploinsufficient and *NF1* bi-allelically inactivated skeletal cells in impaired bone healing is crucial for the development of targeted therapies for CPT patients. Since the *Nf1* mutation leads to deficiencies in many skeletal cell types, we hypothesise that computational models are a suitable tool to investigate the influence of these deficiencies on the bone healing outcome^11^,^12^. Furthermore, a computational model allows the investigation of both individual as well as combined effects of altered cellular phenotypes, something that is near impossible to achieve experimentally. Hence, this study will use a well-established multiscale computational model of normal bone regeneration^13^,^14^,^15^,^16^ to investigate the impaired bone healing associated with *Nf1*, in particular the formation of a pseudarthrosis after excessive bowing and pathological fracture, and to improve our fundamental understanding.

## Materials and Methods

The multiscale computational model of bone regeneration was established previously and has been described in detail elsewhere^14^,^17^. In brief, at the tissue level the various key processes of bone regeneration are captured with 10 partial differential equations (PDEs) of the taxis-reaction-diffusion type which describe the evolution in time and space of the mesenchymal stem cell density (*c*_*m*_), fibroblast density (*c*_*f*_), chondrocyte density (*c*_*c*_), osteoblast density (*c*_*b*_), fibrous matrix density (*m*_*f*_), cartilaginous matrix density (*m*_*c*_), bone matrix density (*m*_*b*_), generic osteochondrogenic growth factor concentration (*g*_*bc*_), vascular growth factor concentration (*g*_*v*_) and oxygen tension (*n*). Note that only one generic osteochondrogenic growth factor (*g*_*bc*_) is included, which represents the effect of multiple growth factors present in the fracture callus. The influence of this generic growth factor on differentiation is either chondrogenic or osteogenic depending on the local oxygen tension.

**Figure 1:**
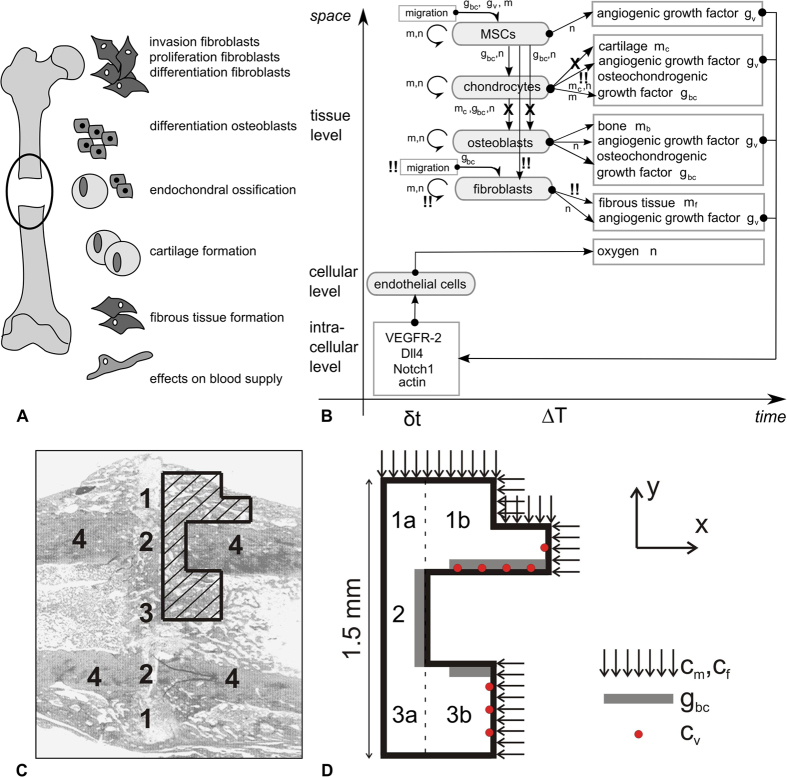
Schematic representation, geometrical domain and boundary conditions of the computational model. (**A**) Schematic illustration of the cellular basis contributing to poor bone repair (adapted from^1^). (**B**) Scale separation map indicating schematically the modelled processes at different spatial and temporal scales. *m* = *m*_*f*_ + *m*_*c*_ + *m*_*b*_ represents the total tissue density. The intracellular variables govern the endothelial cell (EC) behaviour. At the tissue scale, cells can migrate (only mesenchymal stem cells (MSCs) and fibroblasts), proliferate (circular arrows), differentiate (vertical arrows), produce growth factors and extracellular matrix. Blood vessels are a source of oxygen which influences proliferation, differentiation and hypoxia-dependent angiogenic growth factor production. Variables influencing a tissue level process are indicated next to the corresponding arrow. The processes that are down- and upregulated due to the *Nf1* mutation are labeled with ‘X’ for understimulation and ‘!!’ for overstimulation respectively. (**C**) The geometrical domain models a quarter of the real fracture callus geometry^19^ due to symmetry: 1 periosteal callus; 2 intercortical callus; 3 endosteal callus; 4 cortical bone ends. (**D**) Dirichlet boundary conditions are assumed for the mesenchymal stem cells (*c*_*m*_) and fibroblasts (*c*_*f*_) which are released from the periosteum, surrounding soft tissues and bone marrow^20^, and for the osteochondrogenic growth factor (*g*_*bc*_) which is released from the degrading bone ends and the cortex^21^,^22^. The origin of the coordinate system is placed in the left bottom corner of the geometrical domain.

The formation of the blood vessel network is modelled at the level of the individual endothelial cell with a discrete agent-based approach. The computational model includes three different processes that determine the development of the discrete vascular tree, i.e. sprouting (the formation of a new branch, headed by a “tip cell” or “leader cell”), vascular growth (the extension of the branch due to the growth of “stalk cells” or “follower cells”) and anastomosis (the fusion of two branches). The sprouting behaviour of the endothelial cells is derived from the agent-based model of Bentley *et al.*^18^ and is captured with eight intracellular variables, which are unique to every endothelial cell (Fig. 1B). The growth of a blood vessel is modelled by computing the movement of the corresponding tip cell where the tip cell speed depends on the active VEGFR-2 concentration and the tip cell direction is influenced by chemotactic (angiogenic growth factor) and haptotactic (collagen fibres in the extracellular matrix) signals. When a tip cell encounters another blood vessel or when it migrates outside the geometrical domain (illustrated in Fig. 1C–D), an anastomosis is formed during which the leading endothelial cell loses its tip cell phenotype. The newly established connection between the vessels allows for blood flow and the delivery of oxygen and nutrients. Only the endothelial cells that are part of a vascular loop are sources of oxygen.

The geometrical domain of the computational model is deduced from the real callus geometry at three weeks post-fracture in a standardised femoral rat fracture model^19^. We assume a simplified axisymmetrical representation of the geometry so that only a quarter of the domain is simulated (Fig. 1C–D). The parameter values are derived from extensive literature reviews where possible and estimated if no relevant data was available^13^,^14^,^15^,^16^. Both the model variables and the parameter values were non-dimensionalised using proper scaling factors and will be described in their non-dimensionalised form throughout this work^13^,^14^,^15^,^16^. After non-dimensionalisation, the system was also complemented by suitable initial and boundary conditions to ensure the existence, uniqueness and non-negativity of the solution (Fig. 1D).

To examine the effect of the *Nf1* mutation on bone fracture healing, the parameter values of the factors describing the aberrant cellular behaviour of *Nf1* haploinsufficient and *Nf1* bi-allelically inactivated cells were altered (Table 1). A wide range of parameter values (Nf1 range) was investigated to account for the variable proportion of *Nf1* haploinsufficient and *Nf1* bi-allelically inactivated cells in the pseudarthrosis region. In particular, a larger deviation of the parameter values from the normal case represents a higher proportion of bi-allelically inactivated cells. In the current study we have investigated the influence of eight factors described in the literature as contributors to the poor fracture healing outcome in CPT. These factors include the increased invasion of fibrous lesion cells (*c*_*f,BC*_)^1^, increased fibroblastic proliferation (*A*_*f*0_)^1^, increased fibroblastic differentiation (*F*_4_)^23^, reduced osteogenic differentiation (*Y*_11_)^1^,^9^, reduced endochondral ossification (*Y*_3*,cb*_)^1^, reduced cartilage formation (*P*_*mc*_)^1^, increased fibrous tissue formation (*P*_*mf*_)^23^ and increased angiogenic growth factor production (*G*_*gvc*_)^1^,^10^.

**Figure 2:**
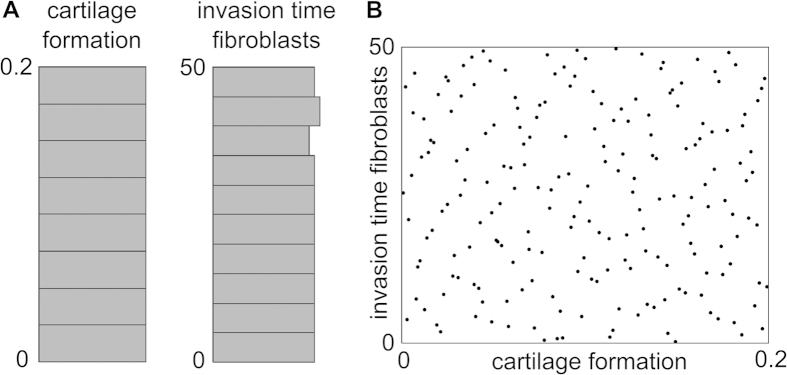
Uniformity of the distribution in a uniform design. (**A**) Histogram showing the distribution of the parameter rate of cartilage formation (*P*_*mc*_) and the invasion time of fibroblasts (*c*_*f,BC*_) in a uniform design. In this particular example, it can be seen that the distribution of the cartilage formation rate (*P*_*mc*_) is uniform, while for the invasion time of fibroblasts (*c*_*f,BC*_), a smaller number of high values are included in the uniform design. (**B**) Scatterplot of all parameter combinations. The parameter combinations are spread out over the entire parameter space.

In order to determine the (relative) importance of the eight factors listed in Table 1, a large sensitivity analysis using “Design of Experiments” (DOE) was conducted^24^,^25^. DOE (or experimental design) is a statistical tool that generates an array of combinations of different parameter values within a predefined parameter space. Next, the computer simulations were performed with these parameter combinations, utilizing high-performance computational resources provided by the VSC (Flemish Supercomputer Center). Finally the results were statistically analysed. Since the computational model of bone regeneration is highly non-linear and uniform designs also cope well with the addition and removal of runs, a uniform design was chosen for the sensitivity analysis. The parameter space (Table 1, Nf1 range) was determined by varying the parameter values of the eight factors mostly in one direction with respect to the normal case, in this way biasing the DOE design to a CPT phenotype. Using the parameter ranges listed in Table 1 (Nf1 range), a uniform design with 200 runs, i.e. 200 parameter combinations, was generated (see also supplementary material). The space-filling design evenly spreads out the parameter combinations over the eight-dimensional parameter space which can be checked with scatterplots and histograms (Fig. 2).

The responses (i.e. model outcomes) that were studied in the sensitivity analysis are listed in Table 2 (see also supplementary material). In order to calculate the tissue fractions, the spatial images were first made binary using tissue-specific thresholds (0 means that the tissue is not present and 1 means that the tissue is present in a grid cell). Subsequently, an equal weight was assigned to the different tissues, i.e. if a grid cell contained three tissues, the area of that grid cell was divided by three in the final calculations of the tissue (area) fractions. Since a pseudarthrosis is defined by the tissues present in the fracture gap area (1a-2-3a in Fig. 1D), all the responses were calculated for this area. An additional response “CI” (complication index) was introduced to assess the degree of severity of CPT (Equation 1). More specifically, the CI (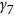) combines two continuous responses (i.e. the amount of fibrous tissue 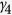 and fibroblasts 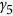, each varying between 0 and 1 for a low and high amount respectively) with a Boolean response 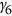 (i.e. bony union or non-union represented by 0 or 1 respectively):


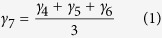


A typical parameter combination for which the value of the CI is small is one or which the degree of severity of CPT is small, or in other words, when the fracture healing proceeds fairly normally. Conversely, a typical parameter combination for which the value of the CI is large, is one for which the degree of severity of CPT is large, or in other words, when the fracture healing is severely impaired.

**Figure 3:**
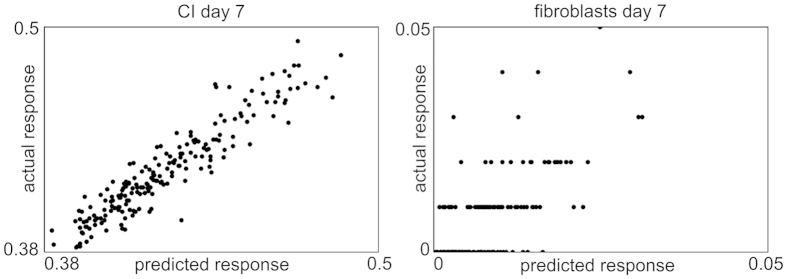
Actual versus predicted plot for the CI response (left) and the fibroblast density (right) on Day 7. The horizontal axis shows the value of the output predicted by the Gaussian process and the vertical axis shows the actual output value of the computational model.

**Figure 4:**
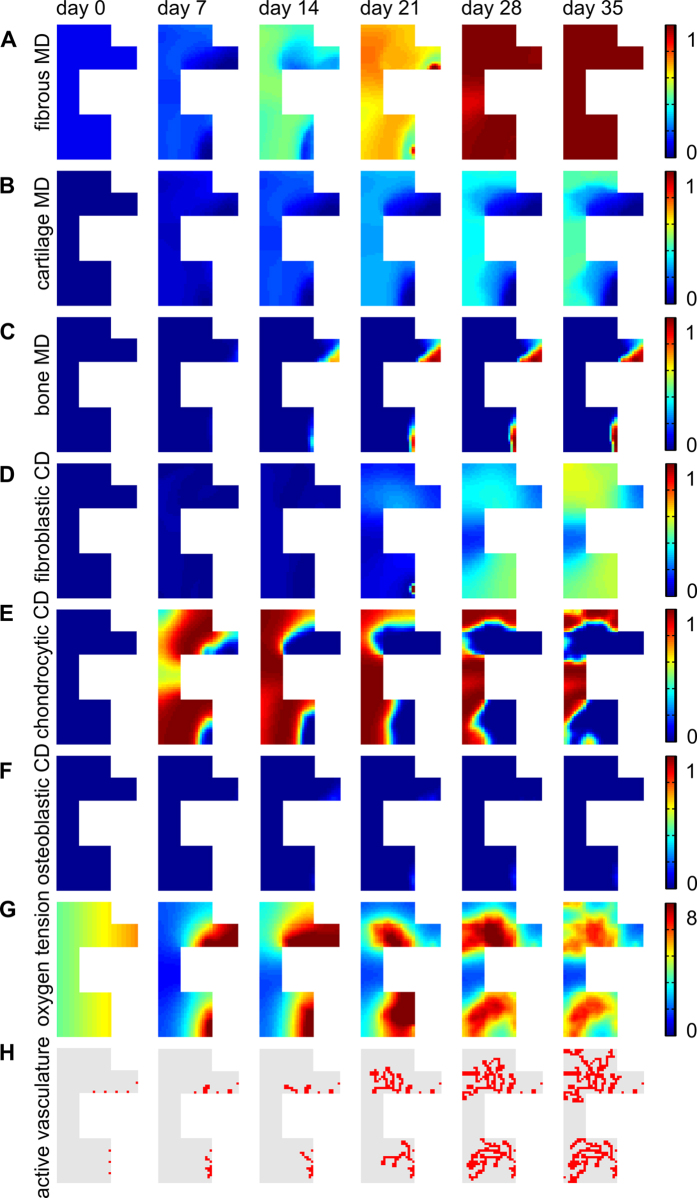
The spatiotemporal evolution of fracture healing associated with Nf1 as predicted by the computational model with the parameter set defined in Table 1 (Nf1 case). (**A**) fibrous MD (×0.1 g/ml), (**B**) cartilage MD (×0.1 g/ml), (**C**) bone MD (×0.1 g/ml), (**D**) fibroblastic CD (×10^6^ cells/ml), (**E**) chondrocytic CD (×10^6^ cells/ml), (**F**) osteoblastic CD (×10^6^ cells/ml), (**G**) oxygen tension (×1%), (**H**) active vasculature. MD = matrix density, CD = cell density. Similarly to previous work, the various tissues (i.e. bone, cartilage and fibrous matrix) and cell types (i.e. fibroblasts, osteoblasts, and chondrocytes) can be present within the same grid cell since their respective densities are constrained independently of the presence of other tissues or cell types^14^,^15^,^16^,^17^.

**Figure 5:**
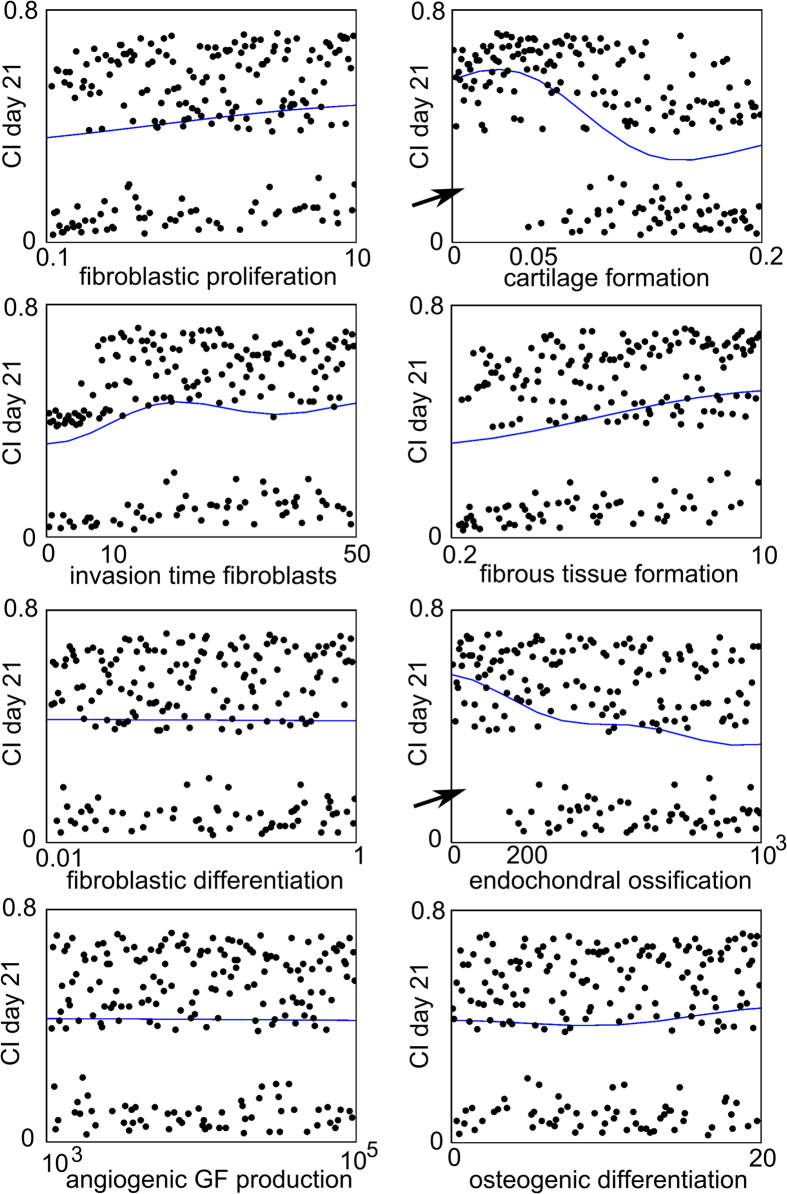
Marginal model plots for *c*_*f_BC*_, *A*_*f0*_, *F*_*4*_, *Y*_*11*_, *Y*_*3cb*_, *P*_*mc*_, *P*_*mf*_ and *G*_*gvc*_. The horizontal axis shows the value of the parameter and the vertical axis shows the value of the CI response on Day 21. The dots indicate the actual results obtained from the computational model (each dot represents the CI response obtained for a particular combination of parameter values in the Nf1 range), and the solid line indicates the results predicted by the Gaussian process. Note that the actual results can be split up in two classes: one for which the CI value is high, resulting in impaired bone healing and one for which the CI value is low. GF = growth factor.

The uniform designs were generated and analysed with JMP (SAS Institute Inc., Cary, North Carolina, USA) using a Gaussian process with a Gaussian covariance function. Unlike an ANOVA-type of analysis, Gaussian processes do not only estimate the importance of individual parameters, but also the influence of the parameters on the outcome of a model. Hence, a Gaussian process will determine the best-fitting statistical model that can predict the model outcome for a certain combination of input parameters (Figs 3, 4, 5). Since the result of a Gaussian process is a statistical model, care should be taken when interpreting the predictions. In order to assess the accuracy of the statistical predictions an actual versus predicted plot generated by JMP is helpful. In this plot the actual results, i.e. the outcomes calculated by the computational model, are compared with those predicted by the statistical model (Fig. 3). In a perfect statistical fit, all the points would be located on a 45-degree line. Fig. 3 (left) shows that the statistical model predicts a good fit for the CI response on Day 7. However, the Gaussian model does not capture the number of fibroblasts on Day 7 well, as illustrated by the large deviations from the 45-degree line in Fig. 3 (right). Since the statistical predictions are accurate for all outcomes except the number of fibroblasts on Day 7 and Day 21 (results not shown), the results on the number of fibroblasts on Day 7 and Day 21 will not be used for further interpretation.

## Results

A non-union resembling a CPT phenotype was predicted by the computational model when all the parameter values were altered according to Table 1 (Nf1 case). Due to the hampered osteogenic and endochondral ossification, a limited number of osteoblasts were formed resulting in a non-union (Fig. 4C–F). The fracture site was however predicted to be filled with chondrocytes and fibroblasts which resulted in the formation of a fibrocartilaginous ‘hamartoma’ (Fig. 4D,E). Although plenty of chondrocytes were present, the amount of cartilage was limited due to the impaired formation of cartilage matrix (Fig. 4B).

The main effects, i.e. the most important factors that influence a specific outcome of the model, are summarised in Table 3. Note that the number of fibroblasts on Day 7 and Day 21 were not well predicted by the statistical model and thus not used for further interpretation. Since the bone tissue fraction was zero on Day 7, the statistical model did not calculate any main effects for this response. The most important factors influencing the defined responses were *P*_*mc*_ (rate of cartilage formation), *A*_*f0*_ (rate of fibroblastic proliferation), *P*_*mf*_ (rate of fibrous tissue formation), *c*_*f_BC*_ (duration of fibroblast invasion) and *Y*_*3cb*_ (rate of endochondral ossification). The factors *F*_*4*_ (rate of fibroblastic differentiation), *Y*_*11*_ (rate of osteogenic differentiation) and *G*_*gvc*_ (rate of angiogenic growth factor production) contributed less to the outcomes of the defined response space. The factor *P*_*mc*_ influenced all outcomes except the amount of fibrous tissue on Day 7 and the CI on Day 7. As a result this factor appeared to be crucial for the fracture healing outcome and was studied in more detail.

**Figure 6:**
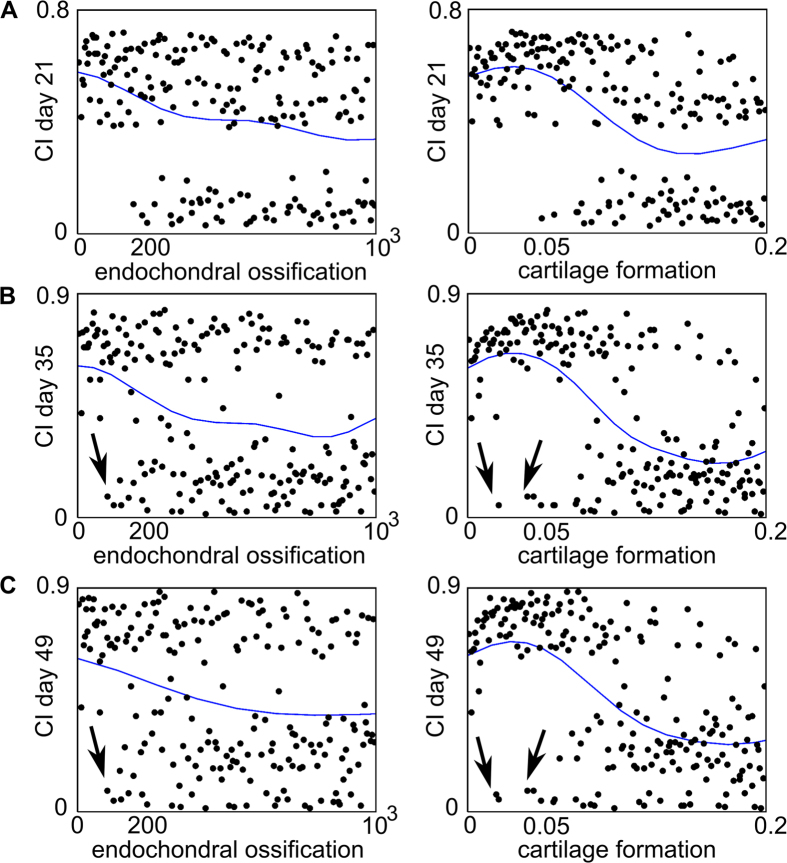
Marginal model plots for *Y*_*3cb*_ and *P*_*mc*_ on Day 21 (A), Day 35 (B) and Day 49 (C). The horizontal axis shows the value of the parameter and the vertical axis shows the CI value. The dots indicate the actual results obtained from the computational model (each dot represents the CI response obtained for a particular combination of parameter values in the Nf1 range), and the solid line indicates the results predicted by the Gaussian process. Note that the actual results can be split up in two classes: one for which the CI value is high, resulting in impaired bone healing and one for which the CI value is low.

In order to study the main effects of the CI response in more detail, marginal model plots are a helpful tool. These plots show the actual and predicted values of the output variable as a function of only one factor when disregarding the influence of other factors. Figure 5 shows the marginal model plots for the CI value as a function of *c*_*f_BC*_, *A*_*f0*_, *F*_*4*_, *Y*_*11*_, *Y*_*3cb*_, *P*_*mc*_, *P*_*mf*_ and *G*_*gvc*_. Firstly, it can be noted from Figs 5, 6 that a wide range of CI values (0.01-0.89) were predicted by the computational model. Secondly, Fig. 5 shows that the dots, indicating the actual values of the CI response, split into two classes, one for which the CI value was high, resulting in impaired healing and one for which the CI value was low. For the factors *c*_*f_bc*_, *A*_*f0*_, *F*_*4*_, *Y*_*11*_, *P*_*mf*_, *G*_*gvc*_, dots were present in both classes, regardless of their respective parameter values. However, for cartilage formation (*P*_*mc*_) values below 0.05 and endochondral ossification (*Y*_*3cb*_) values below 200, the CI value was always high, leading to an impaired bone healing phenotype (Fig. 5, arrows). Therefore, *Y*_*3cb*_ and *P*_*mc*_ discriminated between the two classes (which also corresponded to the results of Table 3).

**Figure 7:**
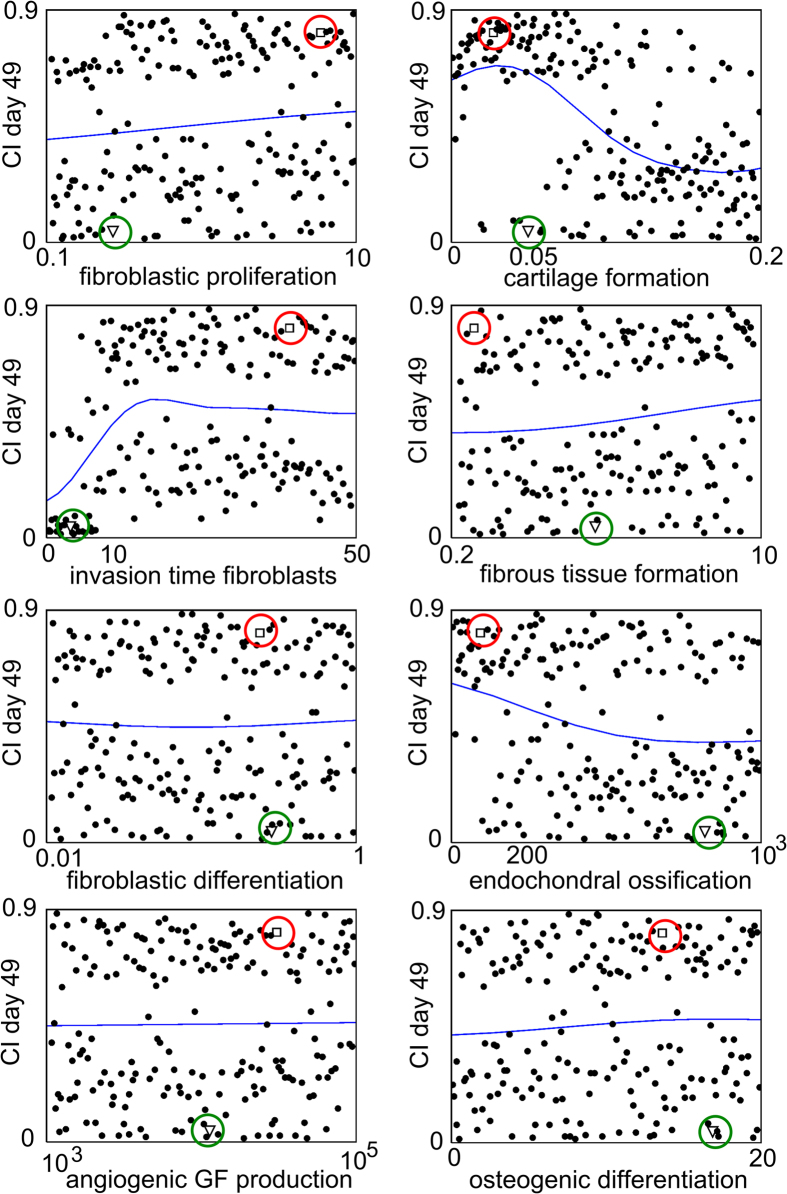
Marginal model plots for *c*_*f_BC*_, *A*_*f0*_, *F*_*4*_, *Y*_*11*_, *Y*_*3cb*_, *P*_*mc*_, *P*_*mf*_ and *G*_*gvc*_. The horizontal axis shows the value of the parameter and the vertical axis shows the CI value at day 49. The dots indicate the actual results obtained from the computational model (each dot represents the CI response obtained for a particular combination of parameter values in the Nf1 range), and the solid line indicates the results predicted by the Gaussian process. Note that the actual results can be split up in two classes: one for which the CI value is high, resulting in impaired bone healing and one for which the CI value is low. The green circles indicate a particular case when a low *P*_*mc*_ value is associated with a low CI value. The red circles indicate a particular case when a low *P*_*mc*_ value is associated with a high CI value. GF = growth factor.

*Y*_*3cb*_ and *P*_*mc*_ also discriminated between the high and low CI values on Day 35 and Day 49 (Fig. 6). However, on Day 35 and Day 49 some low CI values were predicted for *P*_*mc*_ and *Y*_*3cb*_ parameter values below 0.05 and 200 respectively (Fig. 6, arrows). A closer look at the marginal model plots of the CI value on Day 49 showed that the influence of *Y*_*3cb*_ and *P*_*mc*_ can be modulated by other factors (Fig. 7). The green circle shown in Fig. 7 represents a single case where a low CI value was calculated even though the cartilage formation (*P*_*mc*_) value was low. Indeed, the limited cartilage production rate (*P*_*mc*_) was compensated by a relatively high endochondral ossification rate (*Y*_*3cb*_). Furthermore, the duration of fibroblast infiltration (*c*_*f_BC*_) and the rate of fibroblast proliferation (*A*_*f0*_) were low, which resulted in a small number of fibroblasts and a low CI value.

**Figure 8:**
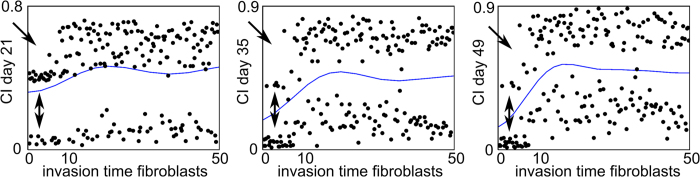
Marginal model plots for *c*_*f_BC*_ on Day 21, Day 35 and Day 49. The horizontal axis shows the value of the parameter, the vertical axis shows the CI value. The dots indicate the actual results obtained from the computational model (each dot represents the CI response obtained for a particular combination of parameter values in the Nf1 range), and the solid line indicates the results predicted by the Gaussian process. Note that the actual results can be split up in two classes: one for which the CI value is high, resulting in impaired bone healing and one for which the CI value is low.

Interestingly, *c*_*f_BC*_ values below 10 led to intermediate values of the CI response (Fig. 8, arrow). The actual dots still belonged to the upper class but did not span the entire range of high CI values. Moreover, the CI values of these simulations were even reduced on Day 35 and Day 49. Consequently, a non-dimensional value of *c*_*f_BC*_ above 10 was imperative for CI values above 0.5 and especially at later time points.

## Discussion

In this study we used a computational model of bone regeneration to investigate impaired fracture healing associated with *Nf1*. To the best of the authors’ knowledge, the model is the first in this field and shows that the combination of aberrant processes in skeletal cells, attributed in the literature to the presence of a *Nf1* mutation, may lead to the prediction of a pseudarthrosis. Indeed, the computational model predicts the formation of a non-union (Fig. 4C), a key characteristic of a pseudarthrosis^2^, by altering the parameter values of eight key factors. Furthermore, the predicted tissues are highly reminiscent of a hamartoma, including a large quantity of fibrous tissue, some cartilage and limited endochondral ossification^26^ (Fig. 4A–C).

Fibroblasts are predicted to be the major cell type present in the hamartoma (Fig. 4D). However, Cho *et al.* measured fibrous hamartoma cells at different stages of osteoblastic differentiation while maintaining some mesenchymal lineage phenotype^27^. These findings are also supported by the results of Lee *et al.* that show an aberrant growth of Nf1 haploinsufficient periosteal cells which have failed in terminal osteoblastic differentiation^28^. It is further hypothesised that the fibrous hamartoma, a key characteristic of CPT, originates from these compromised cells^9^,^28^. The discrepancy between the predicted and experimentally measured hamartomatous cell type can be explained by the fact that the computational model only describes four distinct cell types: mesenchymal stem cells, fibroblasts, chondrocytes and osteoblasts. Both chondrogenic and osteogenic differentiation require specific stimuli such as growth factors and specific ranges of oxygen tension whereas fibroblastic differentiation is set as the default differentiation pathway in the computational model. As a result, cells that fail to commit to the osteogenic or chondrogenic lineage will become fibroblasts and start to produce fibrous tissue.

The relative importance of the eight altered factors to the model outcome was further explored in a large sensitivity analysis using “Design of Experiments” (DOE). As described earlier, DOE (or experimental design) is a statistical tool that generates an array of combinations of different parameter values within a predefined parameter space. Next, the computer simulations are performed with these parameter combinations and finally the results are statistically analysed. If all the defined responses are taken together, the most influential factors are: rate of cartilage formation (*P*_*mc*_), rate of fibroblast proliferation (*A*_*f0*_), rate of fibrous tissue formation (*P*_*mf*_), duration of fibroblast invasion (*c*_*f_BC*_) and rate of endochondral ossification (*Y*_*3cb*_) (Table 3). For the CI value specifically, the main effects are: rate of cartilage formation (*P*_*mc*_), duration of fibroblast invasion (*c*_*f_BC*_) and rate of endochondral ossification (*Y*_*3cb*_). Interestingly, similar CI values can be obtained through many combinations of altered parameter values although the composition and spatial distribution of the tissues within the hamartoma was different for each combination (results not shown). Furthermore, the computational model predicts a wide range (0.01-0.89) of CI values, corresponding to different degrees of severity by which the pseudarthrosis presents. Numerous classifications have been described in the literature^2^, thereby supporting the large degree of heterogeneity of CPT. The Crawford classification^29^ mainly describes the different stages as CPT progresses whereas the Boyd classification^30^ entails a prognostic value and the Apoil 2 stage classification discriminates between the type of pseudarthrosis (i.e. hypertrophic or atrophic). The large range of predicted CI values is remarkable, particularly if the asymmetrical design of the parameter value ranges is taken into account (Table 1). The parameter values of the eight factors were mostly varied in one direction with respect to the normal case, thus biasing the DOE design to a CPT phenotype.

Two classes can be observed in the predicted range of CI values, one for which the CI value is high, resulting in impaired bone healing and one for which the CI value is low, resulting in normal bone healing. A closer look at the marginal model plots of the CI value indicates that the rate of cartilage formation (*P*_*mc*_), the rate of endochondral ossification (*Y*_*3cb*_) and the duration of fibroblast invasion (*c*_*f_BC*_) are the most important determinants of this behaviour which can be explained as follows. In the computational model several aspects of endochondral ossification are combined in one function (*Y*_*3*_), which is dependent on the local oxygen tension, the local osteochondrogenic growth factor concentration and the local cartilage density^13^,^14^,^15^,^16^. The latter is included to account for the hypertrophy of the chondrocytes. More specifically, a critical cartilage density needs to be reached in the computational model before endochondral ossification starts. Since the majority of the bone formation in the computational model happens through endochondral ossification, an impairment of that pathway will severely affect the bone healing outcome. Hence in retrospect, it is not surprising that the rate of cartilage formation (*P*_*mc*_) and the rate of endochondral ossification (*Y*_*3cb*_) are critical determinants for the CI value. On the one hand, a low rate of cartilage formation (*P*_*mc*_) will delay the onset of the critical cartilage density, delaying endochondral ossification and finally resulting in a non-union. On the other hand, a low rate of endochondral ossification (*Y*_*3cb*_) directly slows down the endochondral ossification process. Moreover, the rate of endochondral ossification (*Y*_*3cb*_) and the rate of cartilage formation (*P*_*mc*_) cannot be compensated for in the current parameter space of the experimental design whereas a limited duration of fibroblast infiltration can be compensated by other factors in the computational model, e.g. by a high rate of fibroblast proliferation or differentiation. The CI value is, however, not only affected by the presence of a non-union, but is also determined by the number of fibroblasts and the amount of fibrous tissue. An impaired endochondral ossification process is consequently insufficient to result in a high CI value. This conclusion was also drawn from Fig. 7 (green circle) where a low rate of cartilage formation (*P*_*mc*_) was associated with a low CI value. Indeed, a minimal duration of fibroblast invasion (*c*_*f_BC*_) is necessary for the computational model to result in a sufficient number of fibroblasts and enough fibrous tissue, reminiscent of a hamartoma.

The current computational study has some limitations. Firstly, the computational model used in this study to explore the mechanisms of impaired healing associated with *Nf1* only focuses on the repair phases of fracture healing, i.e. the soft and hard callus phase. As a result, the model does not capture the early inflammatory response nor the bone remodelling phase, respectively preceding and succeeding the repair phase. However, a pathogenic *Nf1* mutation may affect the inflammatory response^31^. It is also known that a pathogenic *Nf1* mutation results in increased osteoclastic activity^32^, leading to an imbalanced bone remodelling process and consequently to fracture reoccurrence. The incorporation of the inflammatory and bone remodelling response will be the subject of a future extension of the computational model.

Secondly, the computational model implicitly assumes that the mechanical environment of the fracture callus is permissive, meaning amongst other things that the fracture is sufficiently stabilised to allow bone formation (either through intramembranous or endochondral ossification). Hence, we do not consider any mechanoregulatory signals in the presented model. Although inadequate mechanical stimulation is not considered to be the primary cause of CPT, mechanical stimulation may affect the healing response. Indeed, the fibrous hamartoma and the pathologically thick periosteum are believed to prevent bony union due to the mechanical interposition and hampered vascularisation^2^. Moreover, Kühnisch *et al.* report diminished mechanical strength and increased micro-porosity in *Nf1Prx1* bone tissue due to increased osteocyte lacuna size^33^. In addition, the muscle compartment might also be reduced in some NF1 cases, which may limit adequate mechanical stimulation^34^.

Thirdly, the computational model is based on experimental data from mouse models since these small animal models are increasingly used in bone healing studies due to their less expensive housing, shorter breeding cycles, well-defined genetic background and available (genetic) methods to study particular molecular mechanisms of action^35^. However, compared to large animals, rodents have a more primitive bone structure without a Haversian system and use resorption cavities for bone remodeling^35^. Moreover, in order to correctly mimic fracture healing in adults, animals of an age with completed bone growth should be used^36^. The ratio of age at growth plate closure and life expectancy is about 20% in humans which is quite comparable to that of mice but markedly differs from that of other species such as rats (30%), sheep (10%) and dogs (5%)^37^. In the rat, growth plates have been reported to remain open for prolonged periods of time, even throughout the normal life expectancy of the animal^37^. Given that in this study we have focused on the genetic causes of CPT, we believe that a comparison with experimental data from genetic mouse models (e.g. *Nf1*_*osx*_^−/−^, *Nf1Prx1* and *Nf1Col1* mice) is reasonable, albeit keeping in mind the differences that exist between murine and human bone healing. Also, the computational model of bone regeneration was used to investigate the wide range of impaired fracture healing phenotypes in the population of *Nf1* subjects rather than focusing on subject-specific effects.

Fourthly, the following three phenotypical criteria were used to assess the degree of severity of CPT: the absence of cortical bridging of the defect, the amount of fibrous tissue and the amount of fibroblasts (Equation 1). Clearly the results of the DOE should be interpreted in light of this definition which puts a lot of importance on the presence of fibroblasts and fibrous tissue. However, the definition of the CI variable can be readily extended once more clinical data becomes available. Finally, as with all statistical tools, care has to be taken when interpreting the results of the DOE analysis. A uniform design of 200 runs was chosen for a balance between statistical accuracy and computational feasibility. Furthermore, the results of the sensitivity analysis are valid within the chosen parameter and response space.

Taking all the computational results together, it can be stated that an impaired endochondral ossification process and an increased infiltration of fibroblastic cells are key elements for the development of a congenital pseudarthrosis in the computational model. These computational observations are supported by several experimental findings that attribute a crucial role to the hampered endochondral ossification process. For example, Kühnisch *et al.* analysed two conditional mouse models (i.e. *Nf1Prx1*, limb knock-out and *Nf1Col1*, osteoblast-specific knock-out) and report defective mineral binding in the proximity of blood vessels due to impaired bone collagen formation^33^. In addition, Kolanczyk *et al.* report a decreased proliferation and premature hypertrophy of the growth plate chondrocytes as well as impaired osteoblast differentiation in the developing limbs of *Nf1Prx1* mice^38^. A cartilage-specific inactivation of neurofibromin resulted in reduced chondrocyte proliferation in the growth plate, delayed endochondral bone formation and increased degradation of chondrocyte extracellular matrix^39^. Similar findings are reported by Kuorilehto *et al.* who have shown that the highly expressed *Nf1* gene acts as a Ras-GAP in the growth plate during endochondral ossification as well as in the mature bone cells^8^. Seitz *et al.* observed a pathological enrichment of the osteoid volume and osteoid surface in the Nf1 biopsies^40^, accompanied by high bone turnover. Since the overactivation of the Ras-MAPK signaling pathway negatively influences endochondral ossification^8^ and osteogenic differentiation^27^, the pharmaceutical inhibition of this pathway may constitute a potential therapeutic strategy. This hypothesis was explored by Ndong *et al.* who report an improved bone healing and increased callus strength in *Nf1*_*osx*_^−/−^ mice that received a MEK inhibitor (i.e. Trametinib) and BMP2 released locally at the fracture site through a nanoparticle delivery method^41^. Similar findings are described by El-Hoss *et al.* who show that MEK inhibition can promote bone formation in combination with rhBMP2 in a murine model of Nf1 pseudarthrosis although the amount of fibrosis was not suppressed^42^. The latter observation corresponds to the computational prediction that an increased infiltration of fibroblastic cells can also be a key element for the development of a congential pseudarthrosis. Future research is however necessary to confirm this preliminary hypothesis through additional experimental validation.

It is to be expected that, since endochondral ossification and cellular infiltration are fundamental bone healing processes, they might also be affected in other multiple bone pathologies. However, we believe that the interpretation of the results in this study should be restricted to congenital pseudarthrosis linked to *Nf1* due the particular combination of altered factors, reported in the literature to be related to *Nf1* induced changes, the bias in the DOE design and the specific definition of the complication index.

## Conclusion

In this study we used a well-established multiscale computational model of bone regeneration to integrate the state-of-the-art experimental knowledge on the effect of the *Nf1* mutation on bone fracture healing. We have shown that by altering the parameter values of eight key factors, attributed in the literature to the presence of a *Nf1* mutation, all the distinctive characteristics of a pseudarthrosis can be predicted, i.e. the formation of a hamartoma including a large quantity of fibrous tissue, some cartilage and limited endochondral ossification. Furthermore, the computational model is able to capture a wide variety of impaired fracture healing phenotypes associated with *Nf1* through different combinations of altered parameter values. A large sensitivity analysis using “Design of Experiments” (DOE) identified that an impaired endochondral ossification process and an increased infiltration of fibroblastic cells are key elements for the development of a congenital pseudarthrosis in the computational model. Hence, the computational model results have added credibility to the experimental hypothesis of a genetic cause (i.e. *Nf1* mutation) for CPT. Future research efforts should be focused on the characterisation of the endochondral ossification pathway in *Nf1* haploinsufficient and *Nf1* bi-allelically inactivated cells and that of the *in vivo* fracture healing microenvironment of CPT patients as well as on the identification of other potential mechanisms of action in order to obtain a full understanding of the etiology of CPT and improve current treatment strategies.

## Additional Information

**How to cite this article**: Carlier, A. *et al.* Capturing the wide variety of impaired fracture healing phenotypes in Neurofibromatosis Type 1 with eight key factors: a computational study. *Sci. Rep.* **6**, 20010; doi: 10.1038/srep20010 (2016).

## Supplementary Material

Supplementary Information

## Figures and Tables

**Table 1:** Parameter values of the factors describing the aberrant cellular behaviour of *Nf1* mutated cells.

Factor	Symbol	Normal case	Nf1 case	Nf1 range
Invasion time fibroblasts	*c*_*f,BC*_	3	20	0–50
Fibroblastic proliferation	*A*_*f*0_	0.1	2	0.1–10
Fibroblastic differentiation	*F*_4_	0.01	0.2	0.01–1
Osteogenic differentiation	*Y*_11_	20	1	0–20
Endochondral ossification	*Y*_3*,cb*_	1000	50	0–1000
Cartilage formation	*P*_*mc*_	0.2	0.02	0–0.2
Fibrous tissue formation	*P*_*mf*_	0.2	2	0.2–10
Angiogenic growth factor production	*G*_*gvc*_	10^3^	5.10^4^	10^3^–10^5^

The parameter values of the normal case were derived from extensive literature reviews where possible and estimated if no relevant data was available as detailed in previous publications^13^,^14^,^15^,^16^. The parameter ranges of the Nf1 case were estimated from the experimental findings described in literature^1^,^9^,^23^,^1^,^10^.

**Table 2:** Analysed responses and their range of possible values.

Response	Symbol	Type	Day 7	Day 21	Day 35	Day 49
Bone tissue fraction	*γ*_*1*_	Continuous	0%–100%	0%–100%	0%–100%	0%–100%
Fibrous tissue fraction	*γ*_*2*_	Continuous	0%–100%	0%–100%	0%–100%	0%–100%
Cartilage tissue fraction	*γ*_*3*_	Continuous	0%–100%	0%–100%	0%–100%	0%–100%
Fibrous tissue	*γ*_*4*_	Continuous	0–1	0–1	0–1	0–1
Fibroblasts	*γ*_*5*_	Continuous	0–1	0–1	0–1	0–1
Non-union	*γ*_*6*_	Boolean	0/1	0/1	0/1	0/1
CI	*γ*_*7*_	Continuous	0–1	0–1	0–1	0–1

**Table 3:** Results of the uniform design. The main effects that explain at least 10% of the variation in the response are listed for each response.

Response	Gaussian covariance function
Fibrous tissue fraction Day 7	*P*_*mc*_(53%)
Bone tissue fraction Day 7	—
Cartilage fraction Day 7	*P*_*mc*_(53%)
Fibrous tissue Day 7	*P*_*mf*_(56%)*, A*_*f0*_(27%)
Fibroblasts Day 7	—
CI Day 7	*P*_*mf*_(48%), *A*_*f0*_(31%)
Fibrous tissue fraction Day 21	*P*_*mc*_(87%)
Bone tissue fraction Day 21	*P*_*mc*_(25%)
Cartilage fraction Day 21	*P*_*mc*_(81%)
Fibrous tissue Day 21	*P*_*mc*_ (35%)*, C*_*f_BC*_ (20%), *P*_*mf*_ (12%)
Fibroblasts Day 21	—
CI Day 21	*P*_*mc*_(36%), *Y*_*3cb*_(12%)
Fibrous tissue fraction Day 35	*P*_*mc*_(77%)
Bone tissue fraction Day 35	*P*_*mc*_ (33%)*, c*_*f_BC*_ (13%), *Y*_*3cb*_(12%)
Cartilage fraction Day 35	*P*_*mc*_(34%), *Y*_*3cb*_(12%)
Fibrous tissue Day 35	*P*_*mc*_(34%), *c*_*f_BC*_(30%)
Fibroblasts Day 35	*c*_*f_BC*_ (32%)*, P*_*mc*_(31%), *A*_*f0*_(11%)
CI Day 35	*P*_*mc*_(47%), *Y*_*3cb*_(12%)
Fibrous tissue fraction Day 49	*P*_*mc*_ (66%)
Bone tissue fraction Day 49	*P*_*mc*_(34%)*, c*_*f_BC*_(14%), *Y*_*3cb*_(13%)
Cartilage fraction Day 49	*P*_*mc*_(23%), *Y*_*3cb*_(16%)
Fibrous tissue Day 49	*c*_*f_BC*_(39%), *P*_*mc*_(27%)
Fibroblasts Day 49	*c*_*f_BC*_(24%), *P*_*mc*_(15%)
CI Day 49	*P*_*mc*_(43%), *c*_*f_BC*_(16%)
