# Erratum: Capturing the wide variety of impaired fracture healing phenotypes in Neurofibromatosis Type 1 with eight key factors: a computational study

**DOI:** 10.1038/srep46384

**Published:** 2017-06-30

**Authors:** A. Carlier, H. Brems, J. M. A. Ashbourn, I. Nica, E. Legius, L. Geris

Scientific Reports 6: Article number: 20010; 10.1038/srep20010 published online: 01 29 2016; updated: 06 30 2017.

The original HTML version of this Article listed an incorrect volume number. This has now been corrected in the HTML version; the PDF version was correct at the time of publication.

